# Epigenetics could explain some Moroccan population colorectal cancers peculiarities: microsatellite instability pathway exploration

**DOI:** 10.1186/s13000-015-0326-9

**Published:** 2015-06-24

**Authors:** Mohammed Sekal, Hassania Ameurtesse, Laila Chbani, Karim Ouldim, Sanae Bennis, Mohammed Abkari, Amal Boulouz, Dafr Allah Benajah, Basher Benjelloun, Abdelmalek Ousadden, Khalid Ait Taleb, Said Ait Laalim, Imane Toghrai, Khalid Mazaz, Samia Arifi, Nawfel Mellas, Karima El Rhazi, Taoufiq Harmouch, Sidi Adil Ibrahimi, Afaf Amarti Riffi

**Affiliations:** Department of pathology, University hospital Hassan II of Fez, Fez, Morocco; Department of molecular genetics, University hospital Hassan II of Fez, Fez, Morocco; Department of gastroenterology, University hospital Hassan II of Fez, Fez, Morocco; Department of visceral surgery, University hospital Hassan II of Fez, Fez, Morocco; Department of oncology, University hospital Hassan II of Fez, Fez, Morocco; Department of epidemiology, University hospital Hassan II of Fez, Fez, Morocco

**Keywords:** Colorectal cancer, Microsatellite instability, *BRAF* gene, DNA methylation

## Abstract

**Background:**

Colorectal Cancers (CRC) are one of the most common malignancies in the world. Their incidence in Morocco, between 2005 and 2007, was 5.6 for 100000 inhabitants, which is very low compared to what found in developed countries. In addition, CRCs show a high frequency of rectal localizations, and occurs in a younger population in Morocco compared to what found in developed countries.

The purpose of this study is to confirm these CRC peculiarities in Morocco and try to explain them by exploring the microsatellite instability molecular pathway.

**Methods:**

This is a prospective observational study conducted since January 2010, including 385 patients admitted in Hassan II University Hospital of Fez. We collected clinical, radiological and pathological data. We investigated the expression of mismatch repair (MMR) proteins in 214 patients and *BRAF* gene mutations in 159 patients.

**Results:**

Mean age was 55.08 +/− 15.16 years. 36.5 % of patients were less than 50 years old and 49.3 % of tumors were localized in the rectum. Loss of MMR protein expression was observed in 11.2 % of cases. It was independently associated with individual or family history of cancer belonging to Hereditary Non-Polyposis Colorectal Cancer (HNPCC) spectrum (p = 0.01) and proximal localization (p = 0.02). No *BRAF* mutation was detected in all cases.

**Conclusions:**

These results confirm the high occurrence of CRCs to young patients and the high frequency of rectal localizations in Moroccan population. They mostly show an absence of BRAF mutation, supposing a rarity of *MLH1* promoter hypermethylation pathway, which may even partially explain the CRC peculiarities in our context.

**Virtual Slides:**

The virtual slide(s) for this article can be found here: http://www.diagnosticpathology.diagnomx.eu/vs/5868184711716884

## Background

Colorectal cancers (CRC) are one of the most common malignancies representing the third most common cancer for men and the second for women in the world [1]. Their incidence in Morocco, between 2005 and 2007, was 5.6 for 100000 inhabitants [2]. Several previous epidemiological studies demonstrated that the CRC incidence in Morocco is similar to that in neighboring Maghreb countries but very low compared to what found in developed countries [3] (Table 1). In addition, CRCs show a high frequency of rectal localizations and occur more to a younger population compared to what found in developed countries [2, 4]. According to the Casablanca Moroccan Region Cancer Registry, the rectal cancer mean age occurrence is 58.1 years for men and 54.2 years for women; and the colon cancer mean age occurrence is 56.8 years for men and 57.2 years for women [2].

**Table 1:** Comparison of estimated CRC incidence rate (for 100,000 inhabitants) in Morocco with that in other countries in 2012 [3]

	Morocco	Tunisia	USA	Canada	France
Men	9 - 16.1	9 - 16.1	16.1 - 32.2	>32.2	>32.2
Women	<4.5	4.5 - 7.6	>21.8	>21.8	>21.8

CRC develop through a multistep carcinogenic process with an accumulation of epigenetic and genetic changes. So for the same histology, the underlying molecular abnormalities can be very different and could explain the wide variation in clinical course and responses to therapies for different patients. Several molecular mechanisms are implicated in CRC carcinogenesis: Chromosomal Instability (CIN), MicroSatellite Instability (MSI) and the CpG Island Methylator Phenotype (CIMP) [5].

CIN pathway is found in about 85 % of sporadic CRC and in familial adenomatous polyposis. It is characterized by loss or gain of chromosome arms, chromosomal translocations or gene amplifications [6]. This ‘traditional pathway’ is the most well characterized which involves the progression of a conventional type adenoma that may acquire mutation or loss of *APC*, mutation of *KRAS* and *TP*5*3*, and chromosomal instability before the occurrence of an invasive adenocarcinoma [7].

MSI pathway is found in approximately 15 % of sporadic CRC and 95 % of Hereditary Non-Polyposis Colorectal Cancer (HNPCC) syndrome [8, 9]. Microsatellites are short repetitive DNA sequences found throughout the tumor genome that are prone to mutations [10]. MSI occurs in the case of DNA mismatch repair (*MMR*) genes inactivation (*MLH1*, *MSH2*, *MSH6* or *PMS2)* secondary to germline or somatic mutations. MMR deficiency leads to DNA replication errors within microsatellites [11].

Two sub-types of CRC MSI-high have been described:

- One resulting from Germline *MMR* mutations associated to HNPCC Syndrome, an autosomal dominant disorder that accounts for ~3 % of all CRC [12].
- The other from a somatic epigenetic event in the promoter of a single DNA mismatch repair gene (*MLH1*) [12]. The later subset is known as sporadic MSI-H colorectal cancer and is also characterized by extensive methylation of multiple gene promoter regions called the CpG island methylator phenotype (CIMP). Approximately 20 % of CRCs are CIMP-high tumors and are thought to arise from serrated polyps [13]. Sporadic MSI-H colorectal cancers frequently exhibit hotspot mutations in the *BRAF* oncogene [14]. *BRAF* encodes a serine/threonine kinase that is an essential component of the RAF/MEK/ERK/MAPK signaling cascade which promotes cellular proliferation and anti-apoptotic effects. In CRC, *BRAF* mutations are located in a hotspot in exon 15 that leads to a V600E single-amino-acid substitution [15]. *BRAF* mutation is considered a marker for the serrated pathway and is found in approximately 10–15 % of CRC, including the majority of those showing CIMP [16]. It is associated with *MLH1* promoter methylation, indicates a sporadic MSI CRC and essentially excludes a diagnosis of HNPCC (Lynch) syndrome [15, 17, 18].

The aim of this study is to confirm the CRC peculiarities in Moroccan population. Then, to explore the micro satellite instability molecular pathway with the hypotheses, which it could explain these CRC peculiarities, particularly the differences in incidence between Morocco and developed countries.

## Methods

This is a prospective observational study conducted since January 2010, including 385 patients with CRC, admitted in Hassan II University Hospital of Fez.

### Ethics statements

Written, informed consent was obtained from each patient involved in this observational study. This research was approved by Hassan II University Hospital Ethics Committee.

### Study Population and Histological Features

We collected clinical and endoscopic data (age, sex, personal and family history and tumor localization), radiological data (extension to adjacent organs, visceral metastasis), and pathological data (Histologic type and differentiation, local invasion, lymph node metastasis, peritoneal carcinomatosis…). Classification of CRC was done according to 2010 World Health Organization criteria for histological type, differentiation and tumor stage.

### Expression of DNA Mismatch Repair Proteins

Immunohistochemical analysis of DNA mismatch repair proteins MLH1 and MSH2 was performed on Ventana Benchmark Ultra automaton in 214 tumor samples. To increase the sensitivity of immunohistochemistry, we also studied the expression of MSH6 and PMS2 by the same technique [12, 19].

Four μm tissue sections from formalin-fixed paraffin-embedded tissue were stained with antibodies against hMLH1 (clone G168-728; Cell marque), hMSH2 (clone G219-1129; Cell Marque), hMSH6 (clone BC/44; Cell Marque), and PMS2 (clone MRQ-28; Cell Marque). Absence of nuclear staining in tumor cells with the presence of positive staining in surrounding cells (lymphocytes or normal glandular cells) was interpreted as a loss of expression of these proteins.

### DNA isolation

One representative tissue section and the corresponding paraffin block were selected. The tumor area was marked on the Hematoxylin eosin-stained slide. The percentage of tumor cells in the marked area and the relative amounts of different histoanatomical components of the tumor were estimated to guarantee a valid tumor cell content. DNA was then extracted from formalin-fixed, paraffin-embedded tissue with the QIAamp DNA mini kit (Qiagen®, Hilden, Germany) following the manufacturer’s instructions. Moreover, the isolated DNA quality and amplified products were determined by optical density (OD_260/280_) measurements (Nanoview®).

### Microsatellite Instability Assay

Microsatellite instability (MSI) was assessed in 7 tumor samples with loss of expression of MMR proteins by the Molecular Pathology laboratory in Bergonie Institute, Bordeaux (France), using a fluorescent multiplex PCR-based method with a set of five mononucleotide markers (BAT25, BAT26, NR21, NR22, NR24). Polymerase chain reaction (PCR) for the various microsatellite markers was carried out only on tumor DNA. Standard PCR conditions were used. Primers were custom ordered with various fluorescent dyes from life technologies®. The analysis of variability in the length of PCR product corresponding to the microsatellite markers was performed on ABI 3100 (life technologies®). Tumors were classified as MSI-high if >2 of the 5 markers are unstable; microsatellite stable (MSS) when there is an absence of instability and MSI-low if only 1 marker is unstable. This latter is considered as a MSS status.

### Detection of *BRAF* Mutations

#### Mutant allele-specific PCR

Tumor DNA was analyzed in 159 CRC to detect the V600E point mutation in exon 15 of the *BRAF* gene by the use of an allele–specific PCR using mutation-specific primers. This PCR was performed in a final volume of 50 μl containing 2 μl of forward primer: 5’-GGTGATTTTGGTCTAGCTACATA-3’, tow μl of reverse primer 5’-GGCCAAAAATTTAATCAGTGGA-3’ (12.5 μMl), 0.4 μl of Taq polymerase Platinium®, 5 μl of Mix 10X Buffer (Invitrogen®), 1 μl of dNTPs (10 mM), 2 μl of MgCl_2_ (50 mM), 32.6 μl of free DNA/RNA water and 50 ng of DNA. Then, an agarose gel electrophoresis was performed for the resulting PCR products with a positive control containing the *BRAF* V600E mutation.

#### PCR- direct sequencing

In addition, for all tumors with loss of expression of MMR proteins, and 6 tumors with conservation of expression of MMR proteins, the result of the *BRAF* allele-specific PCR was confirmed by screening of *BRAF* (exon 15) using direct sequencing of PCR products in forward and reverse. PCR products generated using the following primer pairs: forward 5’-TGCTTGCTCTGATAGGAAAATG-3’ and reverse 5’-GTAACTCAGCAGCATCTCAGGG-3’. This PCR was performed in a final volume of 50 μl, containing 0.4 μl of Taq polymerase Platinium®, 5 μl of Mix 10X Buffer (Invitrogen®), 1 μl of dNTPs (10 mM), 2 μl of forward and reverse primers (12.5 μM), 2 μl of MgCl_2_ (50 mM), 32.6 μl of free DNA/RNA water, and 50 ng of DNA. Thermocycling was performed at 95 °C for 10 min, followed by 35 cycles of 95 °C for 30 s, 60 °C for 60 s and 72 °C for 30 s, and one last cycle of 72 °C for 10 min. The resulting PCR products were purified, and tested for the presence of mutations by bi-directional Sanger sequencing on the 3500 Genetic Analyzer (Applied Biosystems®) using the original PCR primers and the BigDye® Terminator V1.1 Cycle Sequencing Kit (Life technologies, Foster City, CA, USA), according to the manufacturer’s recommendations.

These tests are ongoing for the remaining patients.

### Statistics analysis

The data were analyzed on the software EPI Info version 3.4.

- Firstly, we studied all independent variables distribution of: age, sex, personal or family history of cancer belonging to HNPCC spectrum, histological and immunohistochemical parameters.

- In second time, we compared patients with preserved MMR proteins and patients with loss of MMR protein expression using the chi-square test and Fisher’s exact test. Values were considered statistically significant when p < 0.05.

## Results

### Descriptive study

#### Clinical and radiological features

Mean age was 55.08 +/− 15.16 years. 36.5 % of patients were less than 50 years old. Sex ratio M/F = 1.05.

10.1 % of patients had an individual or family history of cancer belonging to HNPCC spectrum. At diagnosis, the average tumor size was 5.8 +/− 2.5 cm. 49.3 % of tumors were located in the rectum, while the proximal locations accounted for only 19.4 %.

**Fig. 1:**
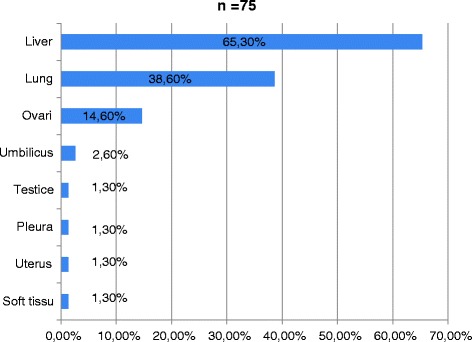
Distribution of metastatic sites at diagnosis

26.3 % of patients presented with one or several visceral metastasis. Secondary localizations were mainly liver, lung and ovary (Fig. 1).

#### Pathological features

Adenocarcinoma was the most common histological type found in 88.1 % of cases. 92.5 % of tumors are well or moderately differentiated. Mucinous component and Independent cells component are found in 16.1 % and 6 % of tumors respectively.

pT3 Stage was the most common found in 78.4 % of cases. Synchronous CRC were present in 3.9 % of cases. Extension to adjacent organs, lymph metastasis and peritoneal carcinomatosis were found respectively at 13.9 %, 29.6 % and 15.6 % of patients (Details in Table 2).

**Table 2:** Clinicopathological and Immunohistochemical Characteristics in CRCs. Fez, Morocco, 2010-2013

Characteristics		Frequencies (%)
*Gender (n = 385)*	Male	51.4
	Female	48.6
*Age rang (n = 385)*	<50 years	36.5
	>50 years	63.5
*Individual or family history of cancer belonging to HNPCC spectrum (n = 208)*	Yes	10.1
	No	89.9
*Localization (n = 385)*	Ascending colon	14.4
	Transverse colon	4.8
	Descending colon	4.8
	Sigmoid	17.9
	Recto-sigmoid junction	9.1
	Upper rectum	6.7
	Through rectum	20.6
	Lower rectum	21.7
*Histologic type (n = 385)*	Adenocarcinoma	88.1
	Mucinous carcinoma	8.6
	Independent cells carcinoma	3.4
*Histological differentiation (n = 385)*	Well	66
	Average	26.5
	Poor	6.8
	Undifferentiated	0.8
*Mucinous component (n = 385)*	Yes	16.1
	No	83.9
*Independent cells component (n = 385)*	Yes	6
	No	94
*Vascular embolus (n = 270)* (*(surgical specimens only)*	Yes	16.1
	No	83.9
*Perineural invasion (n = 270) (surgical specimens only)*	Yes	9.9
	No	90.1
*Synchronous colorectal lesion (n = 359)*	Cancer	3.9
	Single adenoma	11.4
	Multiple adenomas	3.1
	No	81.6
*Stage pT (n = 222) (only surgical specimens without radiochemotherapy)*	1	0.5
	2	12.6
	3	78.4
	4b	8.6
*Lymph node metastasis(n = 270) (surgical specimens only)*	Yes	29.6
	No	60.4
*Stage pN (n = 270) (surgical specimens only)*	0	60.4
	1mic	0.4
	1a	12.6
	1b	10.7
	2a	8.9
	2b	7
*Radiological or/and pathological aspect of extension to adjacent organs (n = 303)*	Yes	13.9
	No	86.1
*Visceral metastasis (n = 285)*	Yes	26.3
	No	73.7
*Peritoneal carcinomatosis (n = 288)*	Yes	15.6
	No	84.4
*Stage M (n = 285)*	0	65.6
	1a	14.4
	1b	20
*Expression of MMR proteins (IHC) (n = 214)*	Loss	11.2
	Conservation	88.8
*MMR proteins not expressed (n = 24)*	MLH1 and PMS2	66.7
	MSH2 and MSH6	33.3

**Fig. 2:**
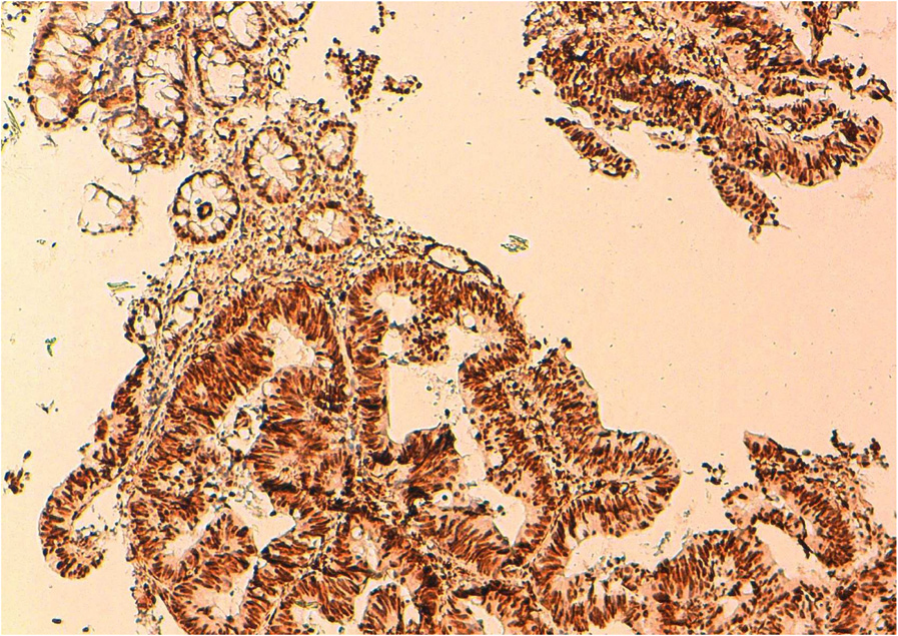
Immunohistochemical study of a colonic adenocarcinoma using anti-MSH2 antibodies (Grossissment x200): Conservation of MSH2 expression both in normal mucosa and carcinoma cells

**Fig. 3:**
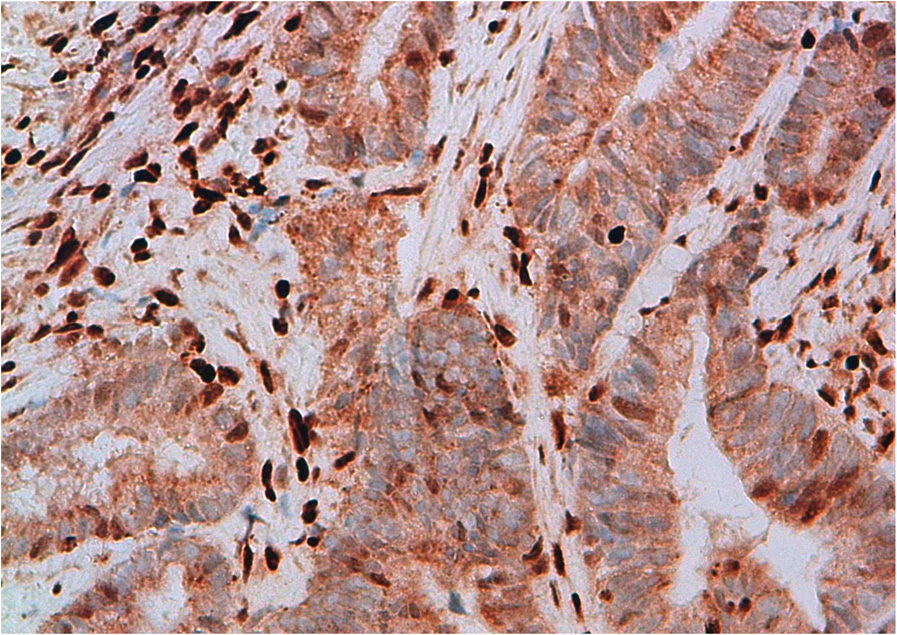
Immunohistochemical study of a colonic adenocarcinoma using anti-MLH1 antibodies (Grossissment x400): Loss of MLH1 expression in tumor cells (Internal control positive)

Loss of MMR protein expression was observed in 11.2 % of cases. It is dominated by the simultaneous loss of MLH1 and PMS2 proteins which represents 2/3 of cases with MMR protein loss (Figs. 2 and 3). Table 3 shows the principal clinical and pathological features of patients with loss of expression of MMR proteins.

**Table 3:** Principal clinical and pathological features of patients with MMR proteins loss. Fez, Morocco, 2010-2013

Cases	Age	Gender	Localization	Histologic type	Histologic Differenciation	Stage pT	Stage pN	Stage M	Lost proteins
1	45	Male	Right colon	Adenocarcinoma	well	2	0	0	MLH1/PMS2
2	45	Male	Right colon	Mucinous Carcinoma	well	3	0	0	MSH2/MSH6
3	49	Male	Rectum	Adenocarcinoma	well	x	x	1a	MLH1/PMS2
4	80	Female	Right colon	Adenocarcinoma	average	3	0	1a	MLH1/PMS2
5	54	Male	Left colon	Mucinous Carcinoma	average	3	0	x	MSH2/MSH6
6	64	Female	Right colon	Adenocarcinoma	well	3	0	0	MLH1/PMS2
7	38	Male	Right colon	Adenocarcinoma	well	3	0	x	MLH1/PMS2
8	61	Male	Right colon	Adenocarcinoma	well	3	1a	x	MLH1/PMS2
9	41	Male	Left colon	Adenocarcinoma	well	x	x	1b	MSH2/MSH6
10	55	Male	Right colon	Adenocarcinoma	poor	3	0	x	MSH2/MSH6
11	48	Male	Right colon	Adenocarcinoma	poor	4a	0	1b	MSH2/MSH6
12	31	Male	Rectum	Adenocarcinoma	average	x	x	1a	MLH1/PMS2
13	71	Male	Left colon	Adenocarcinoma	average	3	2a	1a	MLH1/PMS2
14	70	Male	Rectum	Adenocarcinoma	well	3	2a	x	MLH1/PMS2
15	81	Male	Right colon	Adenocarcinoma	poor	3	0	x	MLH1/PMS2
16	50	Female	Left colon	Adenocarcinoma	average	x	x	1a	MLH1/PMS2
17	56	Female	Right colon	Adenocarcinoma	average	4b	0	1b	MLH1/PMS2
18	50	Female	Right colon	Adenocarcinoma	average	3	0	1b	MLH1/PMS2
19	28	Male	Right colon	Adenocarcinoma	poor	3	0	0	MLH1/PMS2
20	53	Male	Right colon	Adenocarcinoma	well	3	0	0	MSH2/MSH6
21	74	Male	Left colon	Adenocarcinoma	average	3	0	0	MLH1/PMS2
22	53	Male	Right colon	Adenocarcinoma	well	3	0	0	MSH2/MSH6
23	70	Male	Not specified	Adenocarcinoma	well	x	x	x	MSH2/MSH6
24	56	Male	Left colon	Adenocarcinoma	well	4b	0	0	MLH1/PMS2

#### Molecular analysis

**Fig. 4:**
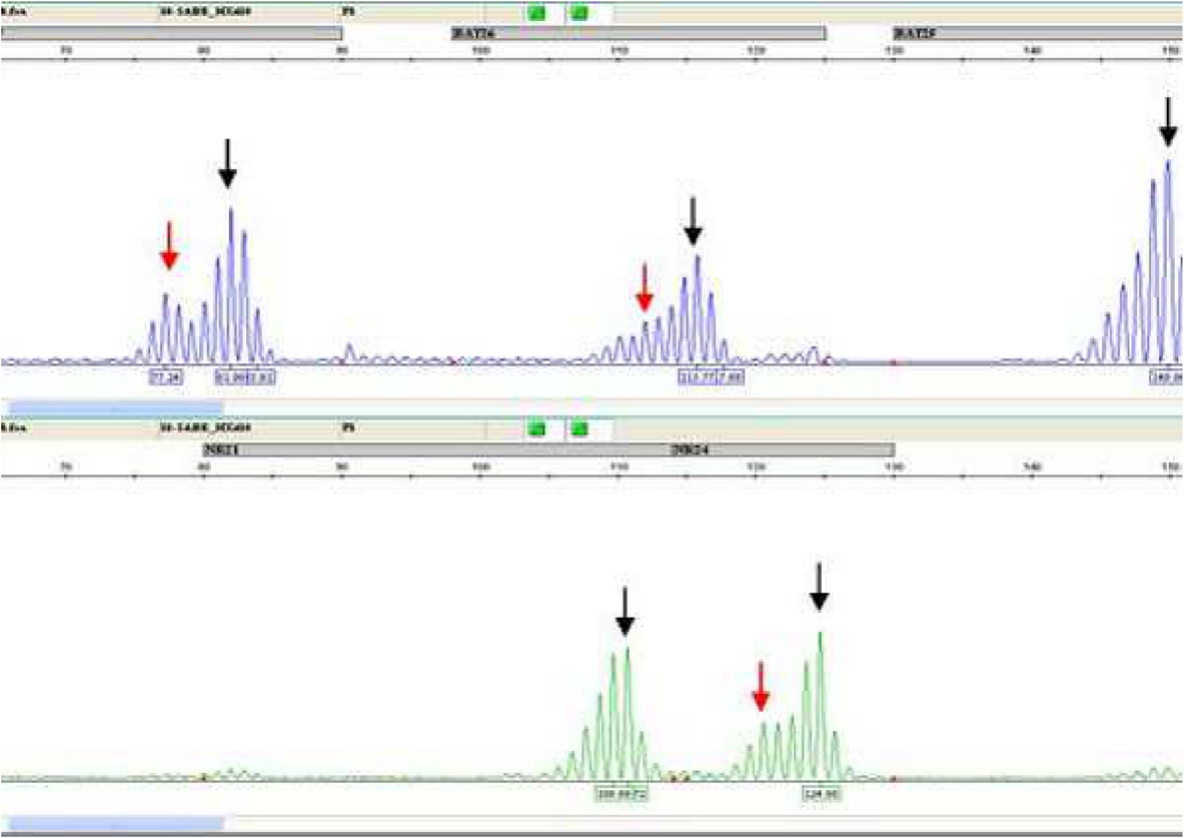
DNA fragment length analysis showing a profile MSI-H: supernumerary peak at 3/5 markers

Microsatellite instability was confirmed in 7 tumor samples using microsatellite instability assay (Fig. 4).

**Fig. 5:**
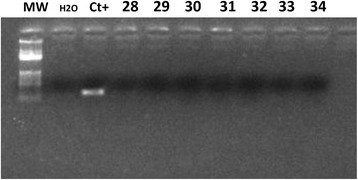
Gel electrophoresis of PCR products of allele specific *BRAF* showing an absence of V600E mutation

**Fig. 6:**
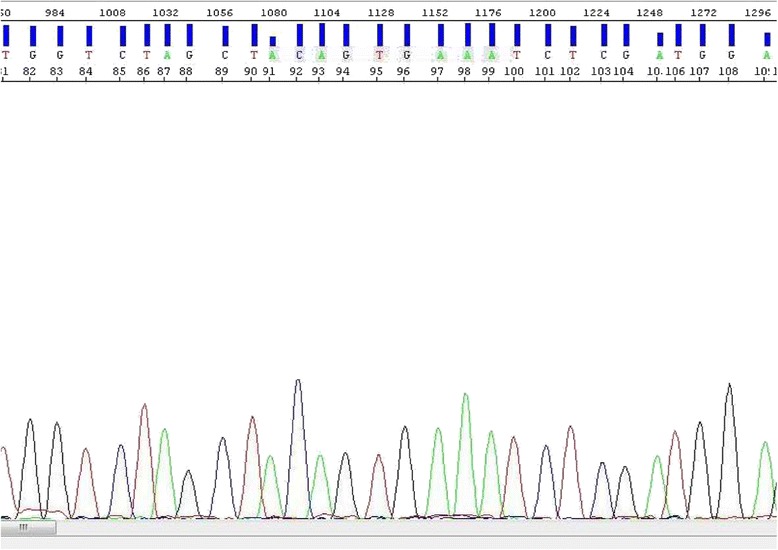
Case Sanger sequencing: no mutation of *BRAF* forward strand

*V600E BRAF* mutation detection was performed on 159 cases, and did not find any mutation (Figs. 5 and 6). This test is in progress for the remaining cases.

Data have been deposited in Cosmic database. Accession Numbers: (unique id_study, 605).

### Analytical study

Univariate analysis demonstrated significant association between loss of MMR proteins with the presence of Individual or family history of cancer belonging to HNPCC spectrum (p = 0.001), Lymph node metastasis (p = 0.02), synchronous colorectal cancer (p = 0.0003) and proximal localization (p = 0.00001). Associations with histologic type, tumor stroma and the presence of visceral metastasis were not statistically significant.

However, in multivariate analysis, only individual or family history of cancer belonging to HNPCC spectrum (p = 0.01, Odds ratio = 35.8 [95 % CI 2.2- 588]); and proximal location (p = 0.02, Odds ratio = 21 [95 % CI 1.4-316.3]) were confirmed to be associated with loss of expression of MMR proteins (Details in Table 4).

**Table 4:** Clinicopathological characteristics in CRCs with MMR proteins expression loss/conservation. Fez, Morocco, 2010-2013

Characteristics	Loss of expression of MMR proteins	Conservation of expression of MMR proteins	Univariate analysis (p)	Multivariate analysis	
				p	Odds ratio
Means Age (years)	55.1	56.4	0.7		
Age rang < 50 years	8/24	60/190	0.48		
Presence of individual or family history of cancer belonging to HNPCC spectrum	4/9	9/190	**0.001**	**0.01**	35.8 [95 % CI 2.2- 588]
Well or moderately differentiated adenocarcinoma	15/24	145/190	**0.07**		
Presence of independent cells component	2/24	13/190	0.52		
Presence of mucinous component	5/24	33/190	0.43		
Presence of perineural invasion	1/19	12/139	0.51		
Presence of vascular embolus	4/19	21/139	0.35		
Presence of lymph node metastasis	3/19	53/138	**0.02**		
Presence of synchronous colorectal cancer	6/23	5/185	**0.0003**		
Presence of radiological or/and pathological extension to adjacent organs	4/21	16/147	0.22		
Presence of visceral metastasis	6/17	31/132	0.21		
Presence of peritoneal carcinomatosis	4/20	21/146	0.35		
***Location***	-	-	**0.00001**	**0.02**	21 [95 % CI 1.4-316.3]
Right colon	14/23	31/184	-	-	
Left colon	6/23	64/184	-	-	
Rectum	3/23	89/184	-	-	

## Discussion

The frequency of *MLH1* promoter methylation in sporadic CRC varied from 0.0 % [20] to 66.9 % [21]. In one Meta-analysis including 5584 patients, the frequency of *MLH1* promoter methylation in unselected CRC was 20.3 % [22]. These cancers with *MLH1* methylation are more commonly observed within older females. It may be due to estrogen decrease with age and could explain why the MSI-H status is more common for women [23].

In our study, we didn’t find any case of CRC with both MMR proteins expression loss and *BRAF* mutation. This result confirms an earlier study led in Hassan II University Hospital of Fez, which took 62 samples of patients with sporadic colorectal adenocarcinomas brought in our pathology laboratory before 2010. So, only one patient (1.6 %) carried *BRAF* codon 600 mutation [24], that is lower than what found in literature, were *BRAF* mutations are found out in approximately 10–15 % of CRC. In Tunisia, where population is similar to Morocco, one study, including 51 patients with CRC, found out also just one patient with a *BRAF* somatic mutation and this tumor has a MSS status [25]. Further analysis of *MLH1* promoter methylation status in the tumor of 4 patients showing MSI-H phenotype and MLH1 protein expression loss with absence of *MLH1* deleterious somatic mutation by Multiplex Ligation-dependent Probe Amplification (MLPA) or sequencing, did not detect aberrant methylation discarding the hypothesis of sporadic cancers due to epigenetic inactivation of *MLH1* gene [25]. Another element that reinforces our results is the rarity of serrated lesions in our context. In an earlier study conducted in our laboratory between 2004 and 2007, we did not find any serrated adenoma among 86 colorectal polyps [26]. On the other hand, the MMR protein expression loss was independently associated in this study with personal or family history of cancer belonging to HNPCC spectrum. This is another index for the high rate of HNPCC syndrome. One study on MLH1 and MLH2 germline mutations is in progress in our laboratory.

MSI-H phenotype is associated with proximal tumor location, poor differentiation and/or mucinous histology, dense lymphocyte infiltration and low frequency of lymph node and distant organ metastasis [10]. In our study, CRCs with MMR protein loss were associated with proximal tumor location. We also found that 49.3 % of CRC are located in the rectum. This confirms data of The Casablanca Moroccan Region Cancer Registry which show that 43.2 % of CRC are located in the rectum [2]. This is a further demonstration of the relative increase in rectal locations for Moroccan population which is very high compared to what is observed in Europe, where it varies between 20 and 35 % [27].

In contrast to colon cancer, rectal cancer missed *V600E BRAF* mutations [28]. Gaedcke and al detected no *V600E BRAF* mutations in 94 rectal cancer patients suggesting that *BRAF* mutations [28] and consequently MLH1 gene promoter hypermethylation [15, 17, 18] do not seem to play any role in rectal cancer pathogenesis. The rarity of *MLH1* gene promoter hypermethylation pathway in Moroccan context could explain the relative high frequency of rectal locations which is in fact caused by a reduction in the number of patients with right location. This would also contribute partially to an overall decrease in the number of CRCs in our context and therefore explain the relative low incidence of CRC.

*MLH1* Promoter hypermethylation is not an isolated process, but part of an overall epigenetic DNA methylation process. For example, in other studies, sporadic MSI tumors were associated with higher rates of hypermethylation of the *PTEN* tumor suppressor *gene* compared to MSS tumors [29]. To explain the probable rarity of *MLH1* promoter hyper methylation in our regional context, we suggest several hypotheses:

1. Some reports noticed that DNA methylation process may be influenced by genetic factors. Genetic variants polymorphisms at a specific locus can influence both regional and distant DNA methylation [30]. Twin and family-based studies suggest that a significant proportion of inter-individual variability in DNA methylation is determined genetically [31]. Relatives of CRC cases with *MLH1* methylation present increased risk of colorectal, stomach and ovarian cancer, suggesting that there may be a heritable factor for CRC and other cancers associated with *MLH1* methylation in non-HNPCC Syndrome CRC [32]. The rarity of hypermethylation in Tunisian patients with CRC [25] could be related to genetic variants. This could also be present within Moroccan population who’s ethnically similar and geographically close to the Tunisian population; and explain the observed absence of *BRAF* somatic mutations.
2. Age is an important risk factor in the development of colorectal cancers [33]. The cause of age-related aberrant methylation is not known. It is possible that upregulation of the de novo methyltransferase DNMT3b could be responsible for the hypermethylation seen in aging [34]. Approximately half of genes which can be involved in age-related methylation are also involved in the pathogenesis of colon cancer, suggesting a role for these genes in the increased cancer susceptibility associated to age [35]. Moroccan population is relatively young compared to developed countries. For example, in 2010, the elderly of more than 60 years was 8.4 % in Morocco [36], against 20.9 % in France [37]. In addition, in 2011, life expectancy at birth in Morocco was 72 years, against 82 years in France and Canada [38]. The relatively low life expectancy in our context may explain the rarity of age related *MLH1* promoter hyper methylation pathway. Youth of our population could also explain the high proportion of young patients, less than 50 years, which is 36.5 % against 6 % in Europe [27].
3. *Epigenetic* drift might be due to the accumulation of small errors in copying DNA methylation marks during successive cell divisions [39]. It has been observed that older monozygotic twin pairs demonstrate greater DNA methylation differences than younger monozygotic twin pairs [40]. The differences between twins were greater for pairs that spent less of their lifetime together or exhibited more different lifestyles suggesting that environmental factors play a role in epigenetic drift [40]. Some carcinogens and environmental factors have been implicated:
4. Cigarette smoke is considered one of the most powerful environmental modifiers of DNA methylation [41]. In a prospective study (n = 37399), cigarette smoking was associated with *BRAF* mutation-positive CRC subtype with a positive dose–response relationship indicating epigenetic modification, which may be functionally involved in smoking-related colorectal carcinogenesis [42]. Carcinogens in cigarette smoke, such as arsenic, chromium, formaldehyde, polycyclic aromatic hydrocarbons, and nitrosamines, can damage DNA by causing double-stranded breaks. Survivor cells display a high capacity for DNA repair. But, the DNA repair sites recruit DNMT1, which methylates CpGs adjacent to the repaired nucleotides [43, 44]. Smoking prevalence is relatively lower in Morocco compared to developed countries. For example, in 2009, the prevalence of smoking among patients over 15 years in Morocco was 33 % for men and 2 % for women, while this prevalence was 36 % for men and 27 % for women in France [38]. This could also explain the rarity of MLH1 promoter hyper methylation pathway in our context.
5. Alcohol is considered one of the factors associated with DNA methylation. Alcohol may induce aberrant DNA methylation patterns alterations; these generally are associated with modulation of the pathways that regulate the availability of S-adenosylmethionine [45]. Indeed, in chronic alcoholics, serum folate levels are significantly reduced compared with healthy subjects. Folic acid deficiency reduces the enzyme S-adenosylmethionine levels [46]. In addition, people with low folate intake/high alcohol intake show a higher frequency of promoter methylation of genes involved in CRC carcinogenesis (e.g., *APC-1A, p14ARF, p16INK4a, hMLH1, O6-MGMT*, and *RASSF1A*) compared with people with high folate intake/low alcohol intake [47]. One meta-analysis including 27 cohort and 34 case–control studies showed a 21 % increased risk of CRC, for moderate drinkers and 52 % increased risk of CRC for heavy drinkers (4 drinks/day) with a dose–risk relationship [48]. Alcoholism prevalence is very lower in Morocco compared to developed countries. For example, in 2008, the prevalence of alcoholism in patients over 15 years was 1.2 % in Morocco, against 12.5 % in France [38]. This could also explain the rarity of MLH1 promoter hyper methylation pathway in our context.
6. Diet has been described as an important factor in colorectal carcinogenesis. Several studies have shown that the highest adherence to Mediterranean diet resulted in a significantly risk reduction for CRC [49, 50]. In a Moroccan cross-sectional survey, 70.1 % of the sample (n = 2214) has a highest adherence to Mediterranean diet [51]. However, a study of this diet impact on CRC associated with MLH1 promoter hypermethylation should be considered to confirm this hypothesis.

Advances in our understanding of colorectal cancer molecular pathology has led to the identification of promising early detection molecular markers for use in non-invasive colorectal cancer screening assays. At this time, stool-based methylated *VIMENTIN* (mVim) is a clinically validated marker for early colorectal cancers detection which is now available in the United States under the name ColoGuard assay (LabCorp®) [52]. In addition, in Europe, there is a blood-based assay that detects methylated *SEPT9* which is being marketed as a colon cancer screening assay under the name Epi *pro*Colon (Epigenomics AG®) [52]. However, the rarity of CIMP-high CRC could make these screening methods, inadequate in our context.

## Conclusions

In Morocco, the rarity of *BRAF* mutation was confirmed in our study that found no cases of CRC with this mutation. MMR proteins expression loss was statistically associated with the presence of Individual or family history of cancer belonging to HNPCC spectrum. These two elements suppose a rarity of MLH1 promoter hypermethylation, likely involved in carcinogenesis of CRC. Further studies are needed to confirm this data which partly explain some peculiarities of this pathology in Morocco, such as the relative high frequency of rectal localizations and low incidence. The relative youth of Moroccan population could also explain the high rate of young patients with CRC.

This rarity of MLH1 promoter hypermethylation pathway in Moroccan population could be explained by the relative low life expectancy or/and the relative low prevalence of smoking and alcoholism or/and the highest adherence to Mediterranean diet.

Lastly, in Morocco and other third world countries, given the adoption in the future of a modern lifestyle and the increase of life expectancy who participate in DNA hypermethylation damage, we consequently expect the continuation of the upward trend in the incidence of this scourge.

## Abbreviations

CRC: Colorectal cancer
DNA: Deoxyribonucleic acid
RNA: Ribonucleic acid
CIN: Chromosomal Instability
MSI: Microsatellite instability
MMR: Mismatch repair
CIMP: CpG Island Methylator Phenotype
HNPCC: Hereditary Non-Polyposis Colorectal Cancer
PCR: Polymerase chain reaction
CGIs: CpG islands
MSS: Microsatellite stable
MLPA: Multiplex Ligation-dependent Probe Amplification
DNMTs: DNA methyltransferases
